# The relationship between emotional intelligence and communication skills in older adults: a cross-sectional study

**DOI:** 10.3389/fpubh.2026.1783872

**Published:** 2026-03-20

**Authors:** Gönül Düzgün, Gökşen Polat, Bugçe Kamer Baybaş Erdoğan, Nurgül Kocakoç, Sevnaz Şahin

**Affiliations:** 1 Department of First and Emergency Aid, Vocational School of Health Services, İzmir Tınaztepe University, İzmir, Türkiye; 2 Elderly Care Application and Research Center, İzmir Tınaztepe University, İzmir, Türkiye; 3 Elderly Care Program, Vocational School of Health Services, İzmir Tınaztepe University, İzmir, Türkiye; 4 Ege University, Bornova, Türkiye; 5 Division of Geriatrics, Department of Internal Medicine, Faculty of Medicine, Ege University, İzmir, Türkiye

**Keywords:** communication skills, emotional intelligence, healthy aging, older adults, psychosocial wellbeing

## Abstract

**Background:**

Population aging has increased interest in psychosocial factors that support healthy aging and social participation. Emotional intelligence is considered a key personal resource that facilitates emotional regulation, social interaction, and communication, yet empirical evidence regarding its role in communication skills among older adults remains limited.

**Objective:**

This study aimed to examine the associations between emotional intelligence and communication skills in older adults.

**Methods:**

This cross-sectional analytical study was conducted with 172 older adults aged 61–85 years enrolled in the University of the Third Age at Ege University between September and December 2025. Data were collected using a sociodemographic questionnaire, the Revised Schutte Emotional Intelligence Scale, and the Communication Skills Scale. Descriptive statistics, non-parametric group comparisons, and Spearman rho correlation analyses were performed.

**Results:**

Participants demonstrated moderate-to-high emotional intelligence levels (mean = 104.63 ± 9.59) and generally good communication skills (mean = 105.90 ± 9.14). Emotional intelligence total scores were positively and significantly associated with overall communication skills (ρ = 0.345, *p* < 0.01). Among emotional intelligence dimensions, optimism showed the strongest and most consistent correlations with all communication skill subdomains (ρ = 0.487, *p* < 0.001). Multiple linear regression analysis showed that emotional intelligence was a significant predictor of communication skills (β= 0.328, *p* < 0.001) after adjusting for age, gender, education, and health status. Age was negatively associated with the use of emotions dimension but positively associated with communication principles, suggesting a compensatory role of experience-based social norms in later life.

**Conclusion:**

Emotional intelligence, particularly optimism, is significantly associated with higher communication skills among socially active older adults. While age-related changes in the use of emotions were observed, the findings suggest that social wisdom may serve as a compensatory mechanism in later life. These results highlight emotional intelligence as a potential psychosocial resource for promoting social engagement and healthy aging, although the cross-sectional nature of the study precludes causal inferences.

## Introduction

The global demographic landscape is witnessing a period referred to as the “silver tsunami,” in which the population aged 60 years and older has, for the first time in history, begun to surpass the younger population. By 2050, the global older population is projected to reach 2.1 billion, with the majority residing in low- and middle-income countries (1). This large-scale aging phenomenon not only places pressure on healthcare systems but also positions the concept of emotional intelligence (EI) as a central focus of research, as it plays a key role in shaping the quality of older adults' social relationships, psychological resilience, and overall quality of life. Emotional intelligence is defined as the ability to recognize, understand, regulate, and use one's own emotions and the emotions of others to guide thinking and behavior (2).

Recent studies have shown that emotional intelligence plays a protective role in the psychosocial adjustment and well-being of older adults. For example, a study published in 2024 found that emotional intelligence levels were positively associated with psychological resilience and life satisfaction, and that higher emotional intelligence helped individuals cope more effectively with challenging life events by reducing perceived stress (3). Another study demonstrated a strong relationship between emotional intelligence and psychological well-being, indicating that older adults with higher emotional intelligence reported higher psychological well-being and life satisfaction (4). These findings suggest that older adults with higher emotional intelligence are more resilient to adverse life conditions and can better buffer the negative effects of stress (3, 4).

Although the aging process has traditionally been portrayed in classical psychology as a period of decline in cognitive abilities—particularly fluid intelligence and processing speed—contemporary gerontological research suggests that emotional functioning is preserved with age and may even improve in several respects. One of the most influential theoretical frameworks explaining this phenomenon is Socioemotional Selectivity Theory. According to this theory, as individuals age, they perceive time as increasingly limited, leading to a motivational shift from knowledge acquisition toward emotionally meaningful goals. Older adults become more selective in their social networks, prioritizing emotionally rewarding interactions and positive experiences. This selectivity results in a focus on deeper, emotionally invested relationships rather than random social contacts, which in turn leads to more refined communication skills within these specific relational contexts (2, 4).

Emotional intelligence influences not only *what* older adults communicate but also *how* they express themselves and *how* they interpret the messages of others. Effective communication requires the synchronized processing of verbal and non-verbal cues. The association between of emotional intelligence and communication operates primarily through empathy, self-regulation, and social awareness. Empirical analyses confirm a positive and significant relationship between all dimensions of emotional intelligence and communication skills (3, 5–7).

In conclusion, enhancing emotional intelligence skills in older adults represents a fundamental strategy not only for improving individual psychological wellbeing but also for strengthening interpersonal communication competence and social support mechanisms. In this context, psychosocial support and educational programs developed for older adults that focus on emotional intelligence are considered effective approaches to improving quality of life and supporting active aging. Accordingly, the present study aims to empirically evaluate the relationship between emotional intelligence levels and communication skills among older adults, thereby addressing a gap in the literature and providing a foundation for practice-oriented recommendations.

## Materials and methods

### Study design

This study was designed as a quantitative, analytical, and cross-sectional investigation conducted to examine the relationship between emotional intelligence on communication skills among older adults. The research was carried out between September 2025 and December 2025 among students enrolled in the University of the Third Age at Ege University, with a total study duration of 6 months. The study was non-interventional in nature and was conducted following approval from the XXX University Medical Research Ethics Committee.

### Study population and sample

The study population consisted of all students enrolled at the University of the Third Age at XXX University. Due to the limited number of eligible participants, a non-probability sampling method was employed, and all students who met the inclusion criteria were invited to participate. A total of 172 individuals were included in the study.

### Inclusion criteria were as follows

- Being between 61 and 85 years of age,
- Being a first- or second-year student at the University of the Third Age,
- Having at least a primary school education,
- Voluntary participation in the study.

### Exclusion criteria were as follows

- Illiteracy,
- Presence of speech or comprehension difficulties,
- Diagnosis of a neurodegenerative disease,
- Being literate without having completed primary school education.

Participants who withdrew from the study or were excluded were followed within the framework of routine procedures.

### Study variables

#### Dependent variables

Revised Schutte Emotional Intelligence Scale (RSEIS): Emotional intelligence was assessed using the Revised Schutte Emotional Intelligence Scale, the Turkish validity and reliability of which were established by Tatar et al. (8). The Cronbach's alpha coefficient was reported as 0.82 for the total scale score and 0.75, 0.39, and 0.76 for the subdimensions. The scale consists of 41 items rated on a 5-point Likert scale (1 = Strongly disagree, 5 = Strongly agree). Negatively worded items are reverse-coded (5 = 1, 4 = 2, 3 = 3, 2 = 4, 1 = 5). The total emotional intelligence score is calculated by summing all item scores. The scale includes three subdimensions: optimism/mood regulation, appraisal of emotions, and social skills/use of emotions. Higher scores indicate higher levels of emotional intelligence (8).

#### Independent variables

Sociodemographic variables: Age, gender, educational level, occupation, living arrangement, year of enrollment at the University of the Third Age, and perceived health status.

Communication Skills Scale: Communication skills were measured using the 25-item Communication Skills Scale developed by Akkuzu and Akkaya (9), which demonstrated a high internal consistency with a reported Cronbach's alpha coefficient of 0.89. The scale is a Likert-type instrument consisting of four subdimensions: communication principles, self-expression, active listening, and initiating communication. Higher scores indicate higher communication skills. The minimum possible score is 25, and the maximum possible score is 125 (9).

### Data collection

Data were collected through face-to-face interviews using a structured questionnaire. The questionnaire consisted of two sections: a sociodemographic data form and the Revised Schutte Emotional Intelligence Scale and Communication Skills Scale. Written informed consent was obtained from all participants, and personal identifying information was kept confidential.

### Hypotheses

H_0_: There is no significant association between emotional intelligence and communication skills among older adults.

H_1_: There is a significant positive association between emotional intelligence and communication skills among older adults.

### Statistical analysis

Data were analyzed using SPSS version 25.0 (IBM Corp., Armonk, NY, USA). Descriptive statistics, including mean, standard deviation, median, minimum, maximum, frequency, and percentage, were used to summarize the data. Normality of distribution was assessed using the Kolmogorov–Smirnov test. For independent group comparisons, Student's *t*-test (two groups) and one-way analysis of variance (ANOVA) (three or more groups) were used for normally distributed variables, while the Mann–Whitney *U* test (two groups) and Kruskal–Wallis *H* test (three or more groups) were applied for non-normally distributed variables. To identify the source of significant differences, Bonferroni-adjusted Mann–Whitney *U* tests were conducted.

Relationships between variables were analyzed using Pearson or Spearman correlation analyses, as appropriate. Furthermore, to identify the predictors of communication skills and to control for potential confounding variables—including age, gender, educational level, and perceived health status—a multiple linear regression analysis was performed. The model was constructed with communication skills as the dependent variable, while emotional intelligence and sociodemographic factors were treated as independent variables. Statistical significance was set at *p* < 0.05.

## Results

### Participant characteristics

A total of 172 older adults were included in the study. The mean age of the participants was 67.60 ± 3.58 years, with a median age of 67.00 years, ranging from 61 to 85 years. Of the participants, 79.7% were women, 52.3% were university graduates, and 54.1% were retired civil servants. It was determined that 40.7% of the participants were living alone. Regarding the renewal year variable, 48.3% of the participants were in their first year. In terms of perceived health status, 61.0% of the participants reported perceiving their health as good (Table 1).

**Table 1:** Sociodemographic characteristics of participants (*n* = 172).

**Variable**	***n***	**%**
**Age (years)**		
Mean ± SD	67.60 ± 3.58	
Median (Min–Max)	67.00 (61–85)	
**Gender**		
Female	137	79.7
Male	35	20.3
**Educational level**		
Primary school	24	14.0
High school	44	25.6
University	90	52.3
University and above	14	8.1
**Occupation**		
Not working	40	23.3
Civil servant	93	54.1
Self-employed	12	7.0
Private sector	27	15.7
**Living arrangement**		
Alone	70	40.7
With spouse	59	34.3
With family (spouse–children)	35	20.3
With child	8	4.7
**Renewal year**		
First year	83	48.3
Second year	89	51.7
**Perceived health status**		
Very good	35	20.3
Good	105	61.0
Moderate	31	18.0
Poor	1	0.6

### Descriptive statistics of emotional intelligence and communication skills

Descriptive statistics for emotional intelligence and communication skills scores are presented in Table 2. The mean total emotional intelligence score was 104.63 ± 9.59, with scores ranging from 80 to 133, indicating generally moderate to high levels of emotional intelligence among participants. Regarding emotional intelligence subdimensions, the mean score for optimism was 48.43 ± 4.96, for use of emotions was 20.12 ± 3.25, and for appraisal of emotions was 36.05 ± 4.96.

**Table 2:** Descriptive statistics of emotional intelligence and communication skills scores.

**Scale/subdimension**	**Minimum**	**Maximum**	**Mean**	**SD**
**Emotional Intelligence Scale**				
Optimism	19.00	60.00	48.43	4.96
Use of emotions	10.00	28.00	20.13	3.26
Appraisal of emotions	18.00	49.00	36.06	4.96
Total emotional intelligence	80.00	133.00	104.63	9.59
**Communication Skills Scale**				
Communication principles	10.00	50.00	43.13	4.67
Self-expression	12.00	20.00	17.63	1.70
Active listening	17.00	30.00	24.67	2.81
Initiating communication	14.00	25.00	20.52	2.42
Total communication skills	77.00	125.00	105.90	9.15

The mean total communication skills score was 105.90 ± 9.14, with scores ranging between 77 and 125, suggesting that participants generally demonstrated good communication skills. When communication skill subdimensions were examined, mean scores were 17.62 ± 1.69 for self-expression, 24.67 ± 2.80 for active listening, 20.51 ± 2.41 for initiating communication, and 43.12 ± 4.66 for communication principles (Table 2).

### Associations between sociodemographic variables, emotional intelligence, and communication skills

Relationships between age and emotional intelligence and communication skills were examined using Spearman's rho correlation analysis. A weak, negative, and statistically significant correlation was found between age and the use of emotions subdimension (ρ = −0.191, *p* = 0.012). In contrast, age was weakly but positively correlated with the communication principles subdimension (ρ = 0.205, *p* = 0.007). No statistically significant relationships were identified between age and optimism, appraisal of emotions, total emotional intelligence, or other communication skills subdimensions and total scores (*p* > 0.05) (Table 3).

**Table 3:** Associations of emotional intelligence and communication skills with sociodemographic variables.

**Variable**	**Optimism**	**Use of emotions**	**Appraisal of emotions**	**EI total**	**Communication principles**	**Self-expression**	**Active listening**	**Initiating communication**	**Communication total**
Age	*ρ, p* > 0.05	ρ = −0.191, *p* = 0.012^*^	*ρ, p* > 0.05	*ρ, p* > 0.05	ρ = 0.205, *p* = 0.007^*^	*ρ, p* > 0.05	*ρ, p* > 0.05	*ρ, p* > 0.05	*ρ, p* > 0.05
Perceived health	KW, *p* < 0.05^†^	KW, *p* > 0.05	KW, *p* > 0.05	KW, *p* > 0.05	KW, *p* > 0.05	KW, *p* > 0.05	KW, *p* > 0.05	KW, *p* > 0.05	KW, *p* > 0.05
Renewal year	MWU = 652.00, *p* = 0.003^*^, *r* = 0.23	*t*_(170)_ = 0.81, *p* = 0.417	MWU, *p* > 0.05	*t*_(169)_ = 1.07, *p* = 0.285	MWU, *p* > 0.05	MWU, *p* > 0.05	MWU, *p* > 0.05	MWU, *p* > 0.05	MWU, *p* > 0.05
Education level	KW, *p* > 0.05	KW, *p* > 0.05	KW, *p* > 0.05	ANOVA, *p* > 0.05	KW, *p* > 0.05	KW, *p* = 0.020^*^^‡^	KW, *p* > 0.05	ANOVA, *p* > 0.05	ANOVA, *p* > 0.05
Occupation	KW, *p* = 0.346	KW, *p* = 0.648	KW, *p* = 1.000	KW, *p* = 0.640	KW, *p* = 0.842	KW, *p* = 0.908	KW, *p* = 0.726	KW, *p* = 0.889	KW, *p* = 0.775
Living arrangement	KW, *p* = 0.028^*^§	KW, *p* > 0.05	KW, *p* > 0.05	KW, *p* > 0.05	KW, *p* > 0.05	KW, *p* > 0.05	KW, *p* > 0.05	KW, *p* > 0.05	KW, *p* > 0.05

KW, Kruskal–Wallis test; MWU, Mann–Whitney U-test; ANOVA, One-Way ANOVA; ρ, Spearman correlation *p* < 0.05.

^†^No significant difference in Bonferroni-adjusted *post hoc* tests.

^‡^Difference between high school and primary school groups.

^§^Difference between spouse and family groups.

Kruskal–Wallis analysis based on perceived health status revealed a statistically significant overall difference in optimism scores (*p* < 0.05); however, no significant differences were identified between groups in Bonferroni-adjusted *post-hoc* comparisons. No statistically significant differences were found for other emotional intelligence or communication skills subdimensions according to perceived health status (*p* > 0.05) (Table 3).

Comparisons based on renewal year indicated no statistically significant differences between groups in total emotional intelligence or use of emotions subdimension scores according to independent samples *t*-test results (*p* > 0.05). Mann–Whitney *U* test results showed that optimism scores differed significantly by renewal year (U = 652.00, Z = −2.99, *p* = 0.003), with first-year participants demonstrating higher optimism than second-year participants. The calculated effect size indicated a small-to-moderate effect (*r* = 0.23). No other significant differences were observed across emotional intelligence or communication skills subdimensions (*p* > 0.05) (Table 3).

Analyses according to education level revealed no statistically significant differences in emotional intelligence subdimensions or total emotional intelligence scores (*p* > 0.05). Within communication skills, a significant difference was identified only in the self-expression subdimension. *Post-hoc* analyses indicated that this difference was attributable to higher self-expression scores among high school graduates compared to primary school graduates (Bonferroni-adjusted *p* = 0.020). No significant differences were found in other communication skills subdimensions or total scores (*p* > 0.05). No statistically significant differences were observed across occupational groups for emotional intelligence subdimensions, total emotional intelligence, communication skills subdimensions, or total communication skills scores (*p* > 0.05). When examined according to living arrangement, a statistically significant difference was found only for the optimism subdimension (*p* = 0.028). Bonferroni-adjusted pairwise comparisons indicated that participants living with family exhibited higher optimism scores than those living with a spouse. No significant differences were identified for other emotional intelligence or communication skills subdimensions (*p* > 0.05) (Table 3).

### Relationship between emotional intelligence and communication skills

Spearman rho correlation analysis was conducted to examine the relationships between emotional intelligence and communication skills. Results indicated that the optimism subdimension was positively and significantly correlated with all communication skills subdimensions and total communication skills score (*p* < 0.001). The strongest correlation was observed between optimism and total communication skills score (ρ = 0.487) (Table 4).

**Table 4:** Correlations between emotional intelligence and communication skills (Spearman rho, *n* = 172).

**Communication skills**	**Optimism ρ (*p*)**	**Use of emotions ρ (*p*)**	**Appraisal of emotions ρ (*p*)**	**EI total ρ (*p*)**
Communication principles	0.449^**^ (< 0.001)	0.027 (0.728)	0.114 (0.138)	0.285^**^ (< 0.001)
Self-expression	0.357^**^ (< 0.001)	0.060 (0.436)	0.051 (0.506)	0.223^**^ (0.004)
Active listening	0.433^**^ (< 0.001)	0.202^**^ (0.008)	0.140 (0.068)	0.321^**^ (< 0.001)
Initiating communication	0.272^**^ (< 0.001)	0.098 (0.200)	0.179^*^ (0.019)	0.258^**^ (0.001)
Communication total	0.487^**^ (< 0.001)	0.090 (0.242)	0.147 (0.056)	0.345^**^ (< 0.001)

ρ, Spearman correlation coefficient *p* < 0.05.

^**^ *p* < 0.01.

The use of emotions subdimension showed a weak but statistically significant positive correlation only with the active listening subdimension (ρ = 0.202, *p* = 0.008). No significant relationships were found between use of emotions and other communication skills subdimensions or total communication skills score (*p* > 0.05).

The appraisal of emotions subdimension was weakly and significantly associated only with the initiating communication subdimension (ρ = 0.179, *p* = 0.019), while no significant relationships were identified with other communication skills subdimensions or total score (*p* > 0.05).

Total emotional intelligence score was positively and significantly correlated with all communication skills subdimensions and total communication skills score (*p* < 0.01), with the strongest association observed between total emotional intelligence and total communication skills score (ρ = 0.345) (Table 4).

To address potential confounding factors and estimate the unique contribution of emotional intelligence to communication skills, a multiple linear regression analysis was performed. The model included age, gender, education level, and perceived health status as control variables. The results indicated that the overall model was statistically significant, *F*_(5, 166)_ = 6.542, *p* < 0.001, accounting for 16.5% of the variance in communication skills (*R*^2^ = 0.165). After adjusting for sociodemographic variables and health status, emotional intelligence remained the only significant predictor of communication skills (β = 0.328, *p* < 0.001). Specifically, for every one-unit increase in emotional intelligence, communication skills increased by 0.312 units, regardless of the participants' age, gender, or education level (Table 5).

**Table 5:** Multiple linear regression analysis predicting communication skills.

**Variable**	***B***	**SE**	**β**	***t***	***p***
(Constant)	58.421	12.145	–	4.810	< 0.001
Emotional intelligence (total)	0.312	0.068	0.328	4.588	< 0.001
Age	0.144	0.182	0.056	0.791	0.430
Gender (female = 1)	0.985	1.420	0.048	0.694	0.489
Education level	0.514	0.522	0.072	0.985	0.326
Perceived health status	0.824	1.115	0.055	0.739	0.461
Model summary	*R*^2^ = 0.165$	*F* = 6.542			*p* < 0.001

B, unstandardized coefficient; SE, standard error; β, standardized coefficient. Dependent variable, communication skills total score.

## Discussion

This study examined the relationship between emotional intelligence and communication skills among older adults and demonstrated the significant association of emotional competencies in shaping the quality of social interactions in later life. The findings indicate that older adults generally exhibit moderate-to-high levels of emotional intelligence (104.63 ± 9.59$) and good levels of communication skills (105.90 ± 9.14). The positive and statistically significant association identified between total emotional intelligence and total communication skills scores (*p* = 0.345, *p* < 0.01$) supports the primary hypothesis of the study and underscores the importance of emotional intelligence as a fundamental psychosocial resource for promoting psychological wellbeing and adaptive communication in older adulthood (3).

### The central role of optimism in interpersonal interaction processes

At the subdimension level, optimism emerged as the factor demonstrating the strongest and most consistent associations with all components of communication skills (ρ = 0.487). This suggests that optimism functions as a facilitating factor against age-related losses and the risk of social isolation. Older adults with a positive emotional orientation may manage interpersonal conflicts more flexibly and utilize social support networks more effectively. Consistent with this, recent international studies highlight optimism as a key protective factor that enhances social functioning, emotional resilience, and overall quality of life in later adulthood (10). In particular, socioemotional selectivity theory highlights that individuals in later life prioritize emotional balance, showing an increased tendency toward positive emotional experiences that foster better social integration (4).

### Opposing age-related trends in emotional functioning and communication principles: the compensatory role of social wisdom

One of the most notable findings is the opposing relationship between age, use of emotions, and communication principles. As age increased, a significant decrease was observed in the “use of emotions” subdimension (*p* = −0.191), whereas the importance attributed to ethical communication principles increased (*p* = 0.205). This pattern suggests that age-related reductions in certain resources may limit the active processing of emotions, while accumulated life experience and heightened sensitivity to social norms compensate for this decline through what may be conceptualized as social wisdom. This compensatory mechanism aligns with systematic reviews indicating that while some cognitive-emotional aspects change, the ability to apply social rules remains a robust asset in older age (3, 11).

Intergenerational communication, which encompasses the exchange of knowledge and emotions across age groups, is essential for social cohesion in modern societies. However, this process is often shaped by age-based stereotypes. Younger adults tend to associate older age with illness, loneliness, weakness, and dependency, while older adulthood is also linked to wisdom, with 76% of young individuals acknowledging that they can learn from older adults' experiences (2, 12).

It is well-established that 60–90% of communication occurs through non-verbal channels. In later life, particularly when verbal expression becomes limited or cognitive decline affects vocabulary, the ability to interpret facial expressions and body language becomes critically important. As Cicero noted nearly two millennia ago, “the face is the mirror of the soul,” and facial expressions serve as a canvas for non-verbal emotional expression (6). Older adults with higher emotional intelligence are more successful in decoding micro-expressions and emotional cues in others, enabling them to anticipate how words or actions may be received and to sustain communication more effectively (13, 14).

### Limits of formal education in emotional intelligence and the role of perceived health

It is important to note that the “Use of Emotions” subdimension demonstrated a low internal consistency in this study (α = 0.39$). This psychometric challenge is consistent with some previous literature suggesting that older adults may perceive and operationalize the “use” of emotions differently than younger populations, potentially due to socioemotional selectivity or cultural differences in emotional regulation. To ensure the robustness of our primary conclusions, we focused on the Total Emotional Intelligence score and the Optimism subdimension, both of which showed acceptable to high reliability in our sample.

The findings indicate that educational level was associated with a significant difference only in the self-expression subdimension, suggesting that formal education is primarily related to specific cognitive and verbal aspects of communication rather than to emotional intelligence as a whole. The literature similarly suggests that the influence of formal education on emotional intelligence is limited and that emotional awareness, regulation, and social interaction skills are more strongly shaped by life experience (15, 16). Studies conducted among university students have shown that although emotional intelligence may be related to academic achievement, age and education level do not consistently predict overall emotional intelligence scores (15, 16). Moreover, professional life experience has been reported to strengthen components such as self-awareness, social skills, and empathy (17).

In this context, the absence of pronounced differences across education, occupation, or household composition in the present sample may be explained by the fact that University of the Third Age students constitute a group that is already engaged in active social learning and life-long development. This supports the view that emotional intelligence is shaped less by formal education and more by lived experience and social interaction.

Although the Kruskal–Wallis analysis revealed a significant overall difference in optimism scores according to perceived health status (*p* < 0.05), *post-hoc* comparisons did not identify specific group-level differences. This may be attributed to limited statistical power resulting from small subgroup sizes, a known limitation of non-parametric *post-hoc* analyses (18). Additionally, perceived health is a subjective construct influenced by personal evaluations, cultural norms, and expectations, which may increase variance and reduce measurement sensitivity.

Nevertheless, the overall trend suggests that individuals who perceive their health more positively tend to utilize emotional resources more effectively and exhibit higher levels of optimism. Previous research highlights strong associations between perceived health, psychological wellbeing, emotional regulation, and optimism, emphasizing that feeling healthy supports stress coping and emotional resilience (19). Accordingly, the findings suggest that perceived health may be indirectly but meaningfully related to the optimism dimension of emotional intelligence and warrant further investigation using larger and more balanced samples.

## Limitations

The findings of this study should be interpreted in light of several limitations. First, the sample consisted exclusively of students enrolled in the University of the Third Age. Compared to the general older population, this group is more socially active, open to interaction, and motivated to learn, which may limit the generalizability of the findings. Future studies should include older adults from diverse socioeconomic backgrounds, including those receiving institutional care or experiencing greater social vulnerability.

Second, the measurement tools used in the study were based on self-report. Subjective variables such as perceived health may be influenced by cultural norms, personality traits, and social expectations, increasing the risk of response bias. Future research incorporating observational methods, qualitative interviews, or multi-source data collection may provide a more comprehensive understanding of emotional intelligence and communication skills.

Third, the cross-sectional design of the study does not allow for causal inferences regarding the relationships between variables. Longitudinal studies are needed to examine how emotional intelligence and communication skills evolve over time in the aging process and to clarify the temporal role of protective factors such as optimism and social wisdom.

Finally, limited subgroup sizes in some non-parametric analyses may have reduced the statistical power of *post-hoc* tests. Future research with larger and more balanced samples would allow for more sensitive detection of subgroup differences.

Furthermore, a significant psychometric limitation was the low internal consistency of the “Use of Emotions” subscale. This low reliability may have limited our ability to detect more nuanced associations involving this specific dimension. Future research should consider using age-adapted emotional intelligence instruments or qualitative methods to better capture how older adults utilize their emotional resources in social contexts.

## Conclusion and recommendations

This study demonstrates that emotional intelligence is a key psychosocial resource shaping communication skills among older adults. The findings indicate that aging is not merely a period of emotional or communicative decline; rather, older individuals are capable of developing experience-based social adaptation and norm-oriented communication competencies. In this respect, the study offers a critical contribution to perspectives that conceptualize aging solely as a passive process of loss.

Optimism emerged as a central emotional resource facilitating communication processes in later life, highlighting the importance of psychological resilience in older adulthood. Although a modest decline in the active use of emotions was observed with increasing age, the growing emphasis on ethical and normative communication principles suggests the activation of a social wisdom-based adaptive mechanism. This indicates that older adults may compensate for emotional limitations through experience and normative sensitivity.

The findings further suggest that emotional intelligence is shaped more by life experience, social interaction, and subjective health perception than by formal education. Therefore, interventions aimed at strengthening emotional and communicative capacities in older adults should prioritize psychosocial resources rather than cognitive performance alone.

Based on the findings, the following recommendations are proposed:

- Educational and support programs for older adults should prioritize modules focused on emotional awareness, optimism, empathic communication, and non-verbal communication skills rather than purely cognitive content.
- Universities of the Third Age and similar lifelong learning programs should provide structured intergenerational interaction environments that make older adults' social wisdom visible and functional.
- Health and social care policies should consider psychosocial interventions aimed at strengthening perceived health as a means of enhancing emotional wellbeing and communication quality.
- Intergenerational communication programs should position older adults not merely as recipients of support but as active agents who transmit experience and contribute to social cohesion.

In conclusion, this study demonstrates that emotional intelligence and communication skills in older adulthood are interrelated, experience-based, and modifiable capacities. Policies and practices aimed at increasing older adults' social participation are likely to produce more sustainable and inclusive outcomes when these emotional and communicative resources are placed at the center.

## Data Availability

The raw data supporting the conclusions of this article will be made available by the authors, without undue reservation.
